# Glucocorticoids Promote the Onset of Acute Experimental Colitis and Cancer by Upregulating mTOR Signaling in Intestinal Epithelial Cells

**DOI:** 10.3390/cancers12040945

**Published:** 2020-04-11

**Authors:** Zhengguo Zhang, Lin Dong, Anna Jia, Xi Chen, Qiuli Yang, Yufei Wang, Yuexin Wang, Ruichen Liu, Yejin Cao, Ying He, Yujing Bi, Guangwei Liu

**Affiliations:** 1 Key Laboratory of Cell Proliferation and Regulation Biology, Ministry of Education, Institute of Cell Biology, College of Life Sciences, Beijing Normal University, Beijing 100875, China; zhangzg226@163.com (Z.Z.); 201731200020@mail.bnu.edu.cn (L.D.); 201831200019@mail.bnu.edu.cn (A.J.); xichen06@hotmail.com (X.C.); 201921200030@mail.bnu.edu.cn (Q.Y.); 201821200044@mail.bnu.edu.cn (Y.W.); 201931200019@mail.bnu.edu.cn (Y.W.); 201721200024@mail.bnu.edu.cn (R.L.); 201921200005@mail.bnu.edu.cn (Y.C.); 201821200008@mail.bnu.edu.cn (Y.H.); 2 Department of Immunology, School of Basic Medical Sciences, Fudan University, Shanghai 200032, China; 3 State Key Laboratory of Pathogen and Biosecurity, Beijing Institute of Microbiology and Epidemiology, Beijing 100071, China

**Keywords:** glucocorticoid, mTOR, colitis, inflammatory bowel diseases, macrophages, neutrophils, epithelial cells, immune cells, microbiota, acute ulcerative colitis, colitis-associated cancer

## Abstract

The therapeutic effects of glucocorticoids on colitis and colitis-associated cancer are unclear. In this study, we investigated the therapeutic roles of glucocorticoids in acute experimental ulcerative colitis and colitis-associated cancer in mice and their immunoregulatory mechanisms. Murine acute ulcerative colitis was induced by dextran sulfate sodium (DSS) and treated with dexamethasone (Dex) at different doses. Dex significantly exacerbated the onset and severity of DSS-induced colitis and potentiated mucosal inflammatory macrophage and neutrophil infiltration, as well as cytokine production. Furthermore, under inflammatory conditions, the expression of the glucocorticoid receptor (GR) did not change significantly, while mammalian target of rapamycin (mTOR) signaling was higher in colonic epithelial cells than in colonic immune cells. The deletion of mTOR in intestinal epithelial cells, but not that in myeloid immune cells, in mice significantly ameliorated the severe course of colitis caused by Dex, including weight loss, clinical score, colon length, pathological damage, inflammatory cell infiltration and pro-inflammatory cytokine production. These data suggest that mTOR signaling in intestinal epithelial cells, mainly mTORC1, plays a critical role in the Dex-induced exacerbation of acute colitis and colitis-associated cancer. Thus, these pieces of evidence indicate that glucocorticoid-induced mTOR signaling in epithelial cells is required in the early stages of acute ulcerative colitis by modulating the dynamics of innate immune cell recruitment and activation.

## 1. Introduction

Acute ulcerative colitis is an acute inflammatory disease of the colon and rectum. The cause of the disease is not very clear. It is urgent and serious. The lesions are mostly seen in the sigmoid colon and rectum and can also extend to the descending colon or even the entire colon [1,2,3,4]. To alleviate acute colitis, treatment with effective drugs and deciding upon selective colectomy in a timely fashion are necessary [1,2,4,5,6]. Acute ulcerative colitis is poorly controlled and often develops into chronic colitis and inflammatory-related cancer, even leading to death [3,7,8,9]. Therefore, the rapid and timely control of acute colitis is an important clinical treatment strategy. At present, glucocorticoids are the first-line drug for colitis treatment [2,3]. If there is no response or exacerbation in 3–5 days, the use of either the immunosuppressive drug cyclosporine (2 mg/kg, intravenous (IV)) or the anti-TNFα antibody drug infliximab (5 mg/kg IV) should be considered [1,3,4,10]. Known controlled evidence supports the use of both treatments. The theoretical basis of combination treatment is generally that the combined use of drugs outperforms the effect of either one alone, but the mechanism is still unclear [3,11]. Even the exact immunoregulatory effect of glucocorticoids in early stage acute ulcerative colitis and whether glucocorticoid therapy is effective during this stage of acute colitis remain controversial [12,13,14,15,16,17,18]. The correct choice of subsequent immunosuppressive drugs is only judged by clinical improvement, but the immunological evidence is still unclear.

Mammalian target of rapamycin (mTOR) is a highly conserved serine/threonine protein kinase. It can form three different complexes in vivo, mTORC1 (mTOR complex 1), mTORC2 (mTOR complex 2) and mTORC3 (mTOR complex 3) [19,20,21,22]. mTOR integrates a series of signaling pathways, such as those related to nutrition, growth factors, energy and stress, and regulates cell metabolism, cell growth, proliferation and survival to play an important role in tumors and other diseases [19]. Increasing evidence [23,24,25] shows that the mTOR signaling pathway plays different roles in pro-inflammatory or anti-inflammatory regulation in different types of immune cell, which are critical for determining the occurrence and development of immune-associated diseases. Recent studies have shown that in mice with colitis and colorectal cancer, the activity of mTOR in inflammatory lesions and tumor lesions is enhanced, and the proliferation of intestinal epithelial cells in inflammatory lesions depends on the activity of mTOR [26,27]. Glucocorticoids usually play a regulatory role through the glucocorticoid receptor (GR) [28]. mTOR also inhibits the anti-inflammatory effect of glucocorticoids in myeloid immune cells [29], but the role of mTOR signaling in the immune regulation of glucocorticoid-treated colitis remains unclear.

In the present study, we sought to determine whether glucocorticoids are effective and how they regulate the onset and course of acute ulcerative colitis, especially in the development of acute colitis with a dextran sulfate sodium (DSS)-induced acute experimental ulcerative colitis mouse model and the effects of glucocorticoids in colitis-associated cancer.

## 2. Material and methods

### 2.1. Mice

All animal experiments were performed with the approval of the Animal Ethics Committee of Beijing Normal University, Beijing, China and of Fundan University, Shanghai, China. C57BL/6 (B6) mice were purchased from the Beijing University Experimental Animal Center (Beijing, China) or Fudan University Experimental Animal Center (Shanghai, China). *mTor*^fl/fl^, *Lyz-Cre* and *Vil-Cre* mice were obtained from the Jackson Laboratory and extensively backcrossed to the C57BL/6 background. Wild-type (WT) controls for mTOR knockout mice (*LyZ-Cre* or *Vil-Cre*; *mTOR*^fl/fl^) included *Cre*^+^ mice (*LyZ-Cre* or *Vil-Cre*; *mTor*^+/+^) to account for the effects of *Cre*. Sex-matched littermates at 7–10 weeks of age were used in the experiments described in this study. All mice were bred and maintained under specific pathogen-free conditions.

### 2.2. Experimental Colitis and Histological Analyses 

Acute experimental ulcerative colitis was induced by feeding the mice drinking water supplemented with 3% DSS daily or the administration of 2.3 × 10^8^ CFU/mouse of *E. coli* O157:H7 (LD_50_) for 5 days, which caused severe erosive colitis, as previously described [30,31]. Body weight and disease activity index (DAI) were assessed on a daily basis. DAI was calculated as previously described [30,32,33], combining weight loss, stool consistency and stool blood content/rectal bleeding. The mice were sacrificed at the indicated time points, and colons were removed for further analysis. 

For colitis histopathological analyses, colons were fixed in 4% paraformaldehyde, embedded in paraffin, cut into 5-μm sections and subsequently stained with H&E, as previously described [33,34,35]. Histological colitis scores were determined as previously described [3,36]. In brief, histological sections were scored as follows: epithelium: normal morphology (0), loss of goblet cells (1), loss of goblet cells in large areas (2), loss of crypts (3) and loss of crypts in large areas (4); infiltration: no infiltrate (0), infiltrate around crypts (1), infiltrate reaching the lamina muscularis mucosae (2), extensive infiltration reaching the lamina muscularis mucosae and thickening of the mucosa (3) and infiltration of the submucosal layer (4). The total histological score represents the sum of both scores and ranges from 0 to 8. For each sample, 10 fields were randomly selected, and the mean grade was calculated.

### 2.3. Flow Cytometry

For the flow cytometry (FCM) analysis of surface markers, cells were stained with antibodies in phosphate-buffered saline (PBS) containing 0.1% (wt/vol) BSA and 0.1% NaN_3_, as described previously [37,38,39]. The following antibodies were purchased from eBioscience (Thermo Fisher, Waltham, MA, USA): anti-CD8 (clone no. 53-6.7; catalog no. #17-0081-82), anti-CD45R/B220 (clone no. RA3-6B2; catalog no. #17-0452-82), anti-CD11b (clone no. M1/70; catalog nos. #17-0112-82 and #11-0112-82), anti-Gr1 (clone no. RB6-8C5; catalog nos. #17-5931-82, #11-5931-82 and #12-5931-82) and anti-CD45 (clone no. 30-F11; catalog nos. #11-0451-82, #17-0451-82 and #12-0451-82). The following antibodies were purchased from BD Biosciences (Lake Franklin, NJ, USA): anti-CD115 (clone no. T38-320; catalog no. #743642), anti-CD3 (clone no. 145-2C11; catalog nos. #553061 and #553066), anti-CD11b (clone no. M1/70; catalog no. #566417), anti-CD45R/B220 (clone no. RA3-6B2; catalog nos. #553088 and #561086) and anti-CD11c (clone no. HL3; catalog no. #560583). The following antibodies were obtained from Biolegend (San Diego, CA, USA): anti-CD11b (clone no. M1/70; catalog nos. #101226, #101224 and #101208), anti-Gr1 (clone no. RB6-8C5; catalog nos. #108417, #108448 and #108418), anti-F4/80 (clone no. BM8; catalog nos. #123116, #123118, #123108, #123110 and #123112) and anti-CD45 (clone no. 30-F11 and catalog nos. #103106, #147708 and #103122). Anti-CXCR2 (clone no. 242216; catalog no. #MAB2164-100) was obtained from R&D Systems (Minnesota, USA). For staining phosphorylated signaling proteins, cells were fixed with Phosflow Perm buffer (BD Biosciences), permeabilized with Phosflow Lyse/Fix buffer (BD Biosciences, Lake Franklin, NJ, USA) and stained with anti-p-S6 (Ser240/244; catalog no. #5364), anti-p-S6 (Ser235/236; catalog no. #14733) and anti-p-mTOR (Ser2448; catalog no. #5536), which were purchased from Cell Signaling Technology (Danfoss, Boston, Ma, USA). Flow cytometry data were acquired on a FACSCalibur (Becton Dickinson, San Diego, CA, USA) or an Epics XL bench-top flow cytometer (Beckman Coulter, CA, USA), and the data were analyzed with FlowJo (TreeStar, San Carlos, CA, USA), as described previously [40]. Cell numbers of various populations were calculated by the multiplication of the total cell number by the percentages of each individual population from the same mouse, followed by averaging. 

### 2.4. Quantitative RT-PCR

Frozen tissue samples were homogenized in ice-cold lysis buffer containing 10 mM HEPES, 2 mM EDTA (pH 8), 5 mM DTT, 1 mM Pefabloc and 1 tablet of mixture of proteinase inhibitors (Roche, Basel, Switzerland). RNA was extracted with a RNeasy kit (Qiagen, Dusseldorf, Germany), and cDNA was synthesized with SuperScript III reverse transcriptase (Invitrogen, Carlsbad, CA, USA). An ABI 7900 Real-time PCR system was used for quantitative PCR, with primer and probe sets from Applied Biosystems (Foster City, CA, USA), as described previously [41]. 

PCR primer sequences are presented in Table 1. The results were analyzed with the SDS 2.1 software. The cycling threshold value of the endogenous control gene (*Hprt1*, encoding hypoxanthine guanine phosphoribosyl transferase) was subtracted from the cycling threshold value of each target gene to generate the change in cycling threshold (DCT). The expression of each target gene is presented as the fold change relative to that of WT unstimulated samples (2–DDCT), as described [40,42].

### 2.5. Quantification of Tissue Cytokine Levels 

Frozen colon samples were homogenized in ice-cold lysis buffer containing 10 mM HEPES, 2 mM EDTA (pH 8), 5 mM DTT, 1 mM Pefabloc, and 1 tablet of a mixture of proteinase inhibitors (Roche, Basel, Switzerland). TNFα and IL-6 concentrations were measured in whole tissue extracts by ELISA according to the manufacturer’s instructions (R&D Systems, Minnesota, USA) and are expressed as picograms per milligram of total protein.

### 2.6. Immunoblot Analysis 

Collected cells were washed twice with PBS and then lysed for 30 min on ice in RIPA solution containing protease inhibitor cocktails (Roche, Basel, Switzerland) and phosphatase inhibitors (Sigma, St. Louis, Missouri, USA), as described previously [43]. Equivalent protein concentrations were subjected to 8%–12% SDS-PAGE (Bio-Rad, Hercules, CA, USA). Anti-p-S6 (Ser240/244; catalog no. #2215), anti-p-S6 (Ser235/236; catalog no. #5316), anti-p-Akt (Ser473; catalog no. #4060), anti-p-AKT (Thr308; catalog no. #13842), anti-GAPDH (catalog no. #5174) and anti-β-actin (catalog no. #58169) were purchased from Cell Signaling Technology (Danfoss, Boston, Ma, USA). HRP-labeled goat anti-rabbit IgG (Invitrogen, Carlsbad, CA, USA) and HRP-labeled goat anti-mouse IgG were used.

### 2.7. Statistical Analysis

All data are presented as the mean ± SD. One-way or two-way ANOVA was used for comparisons among multiple groups with the SPSS software according to the type of data. Student’s unpaired *t* test for comparison of means was used to compare two groups. A *P* value (alpha-value) less than 0.05 was considered to be statistically significant. 

## 3. Results

### 3.1. Dexamethasone (Dex) Treatment Specifically Upregulates mTOR Signaling in Intestinal Epithelial Cells in Acute Experimental Ulcerative Colitis

**Figure 1:**
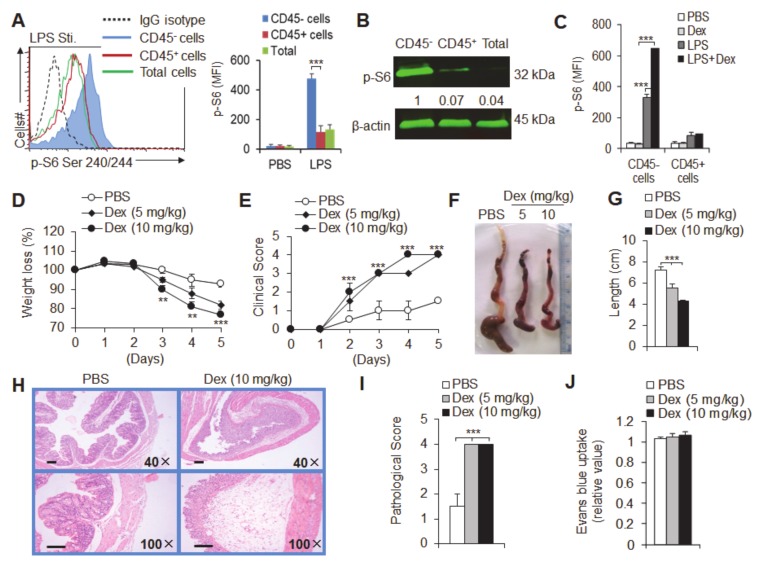
Dexamethasone (Dex) promotes experimental dextran sulfate sodium (DSS)-induced ulcerative colitis. (**A**,**B**) CD45^+^ cells, CD45^-^ cells and total cells isolated from colon tissue, and p-S6 Ser240/244 were determined. A typical histogram of flow cytometry data and the mean fluorescence intensity (MFI) of p-S6 is shown (**A**). Western blot analysis and quantification show the expression of p-S6 Ser240/244. The expression levels of p-S6 Ser240/244 were normalized to the CD45^-^ cell group. The fold changes between p-S6 and β-actin were calculated, and the highest expression (CD45^-^ cells, in this case) was set as 1 (**B**). (C) Mean fluorescence intensity (MFI) of p-S6 Ser240/244 with flow cytometry in CD45^+^ and CD45^-^ cells isolated from colon tissue and treated as indicated. (**D**–**J**) DSS-induced ulcerative colitis mice were intraperitoneally (i.p.)injected with different doses of dexamethasone (Dex; 5 or 10 mg/kg). Weight loss (**D**) and disease clinical scores (**E**) were significantly increased in Dex-treated mice compared to those in phosphate buffer saline (PBS)-treated control mice. Dex-treated mice developed severe ulcerative colitis as shown by colonic shortening (**F**,**G**), severe pathological colitis (**H**) and the pathological score (**I**). (**J**) Basal colonic epithelial permeability was equal in PBS- and Dex-treated mice as measured by Evans Blue uptake. Representative results are based on one of three or four independent experiments performed with similar results. The data are presented as the mean ± SD, (*n* = 4–6 mice per group). Statistical significance was measured by one-way ANOVA. **P< 0.01 and ***P< 0.001, compared with the indicated groups.

mTOR signaling has been shown to play an important role in inflammatory bowel disease [44,45,46,47]. We first observed the differential expression of mTOR signaling in intestinal cells, including intestinal epithelial cells (CD45^+^ cells), intestinal immune cells (CD45 cells) and total intestinal cells. Under inflammatory conditions, lipopolysaccharide (LPS) stimulated the expression of p-S6 Ser240/244 or p-S6 235/236 in intestinal epithelial cells, which was higher than that in intestinal immune cells (Figure 1a,b and Figure S1a). p-S6 is a downstream target of mTOR, and its expression in intestinal epithelial cells was dose-dependent with inflammatory stimulation (Figure S1b). Importantly, glucocorticoid (dexamethasone; Dex) treatment significantly increased the expression of p-S6 Ser240/244 and p-S6 Ser235/236 in intestinal epithelial cells under inflammatory conditions (Figure 1c and Figure S1c). Furthermore, we established a DSS-induced mouse acute colitis model and found a similar result. Acute experimental ulcerative colitis was induced by treating animals with 3% DSS for 5 days. The expression of mTOR in intestinal epithelial cells was higher than that in intestinal immune cells, and Dex treatment significantly increased the expression of mTOR in intestinal epithelial cells in DSS-induced colitis (Figure S2a–c). Moreover, *E. coli O157*-infected colitis was used to better simulate colitis caused by actual acute pathogenic microorganism infection, and we found that the results were consistent (Figure S2d). Taken together, these results strongly suggest that glucocorticoids are helpful for mTOR signaling expression in intestinal epithelial cells and are involved in the regulation of mTOR signaling in the early stages of acute ulcerative colitis.

### 3.2. Dex Treatment Potentiates Acute Experimental Ulcerative Colitis

To investigate the role of glucocorticoids in the early stage of acute experimental colitis, experimental acute DSS mouse ulcerative colitis was induced and treated with Dex at different doses (5 and 10 mg/kg) intraperitoneal (i.p.) injection daily. Mice that were treated with phosphate-buffered saline (PBS) displayed a delayed onset and markedly milder course of disease than Dex-treated mice, which showed symptoms of severe ulcerative colitis (Figure 1d,e). This was evidenced by the significantly reduced body weights and increased DAIs of Dex-treated colitis mice with different doses. Macroscopic and histological analysis after 5 days of DSS treatment revealed severe destructive colitis in Dex-treated mice, as reflected by reduced colonic shortening (Figure 1f,g) and severe histological changes (Figure 1h,i). These results suggest the important role of glucocorticoids in the establishment of acute mucosal inflammation.

Since increased epithelial permeability is critical for the establishment of DSS-induced colitis, we also compared the basal colonic permeability, as measured by Evans blue uptake, and showed comparable alterations (Figure 1j).

### 3.3. Dex-Treated Mice Had Increased Mucosal Immune Cell Infiltration

**Figure 2:**
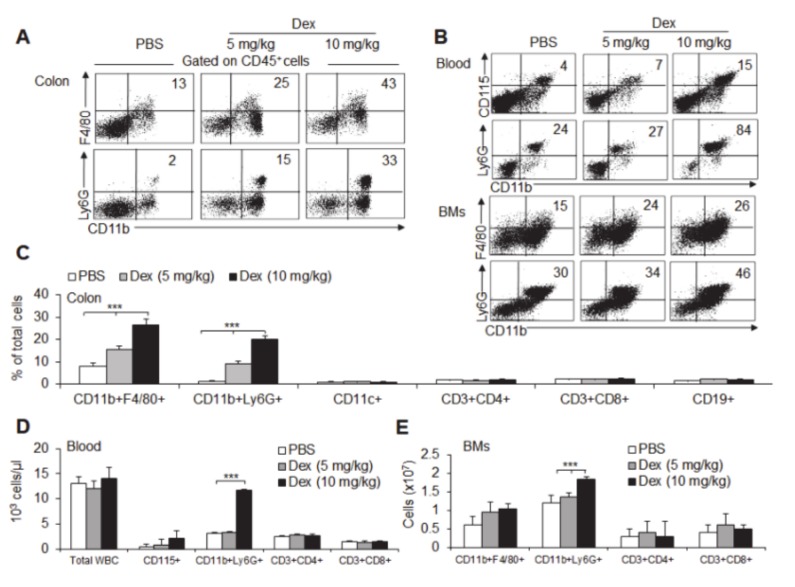
Dex promotes neutrophil and macrophage recruitment in acute ulcerative colitis. The relative proportions of macrophages (CD11b^+^F4/80^+^), neutrophils (CD11b^+^Ly6G^+^), dendritic cells (DCs) (CD11c^+^), CD4^+^ T cells (CD3^+^CD4^+^), CD8^+^ T cells (CD3^+^CD8^+^) and B cells (CD19^+^) in single-cell suspensions derived from mice treated with 3% dextran sulfate sodium (DSS) for 5 days in combination with dexamethasone (Dex) or vehicle (phosphate buffer saline; PBS), with daily intraperitoneal (i.p.) injection. (**A**,**C**) Single-cell suspensions of colonic tissue from mice were isolated. Representative flow cytometry figures are shown (**A**), and the data are summarized (**C**). The blood cells of the mice were analyzed. The CD11b^+^CD115^+^ cells were used as a marker of monocytes. A representative flow cytometry figure is shown (**B**, upper), and the data are summarized (**D**). The bone marrow cells of mice were analyzed, and a representative flow cytometry figure is shown (**B**; lower); the data are summarized (**E**). Representative results are based on one of three or four independent experiments performed with similar results. The data are presented as the mean ± SD (*n* = 4–8 mice per group). Statistical significance was measured by one-way ANOVA. ***P< 0.001, compared with the indicated groups.

To characterize the cell populations involved in mediating acute colitis, we detected the infiltrating mucosal cells with flow cytometry. Five days after the induction of DSS acute experimental colitis, the proportion of intestinal CD45^+^ immune cells was significantly increased in Dex-treated mice (16% vs. 31%). In addition, mice treated with different doses of Dex displayed significantly increased percentages of CD11b^+^F4/80^+^ macrophages and CD11b^+^Ly6G^+^ neutrophils in the colon (Figure 2a). Consistently, a significantly increased percentage of macrophages and neutrophils accumulated in the blood and bone marrow (BM) (Figure 2b). Furthermore, macrophages (CD11b^+^F4/80^+^) and neutrophils (CD11b^+^Ly6G^+^) represented the predominant mucosal cell population in the colon, blood and BM from Dex-treated colitis mice (Figure 2c–e). These results show that Dex treatment significantly potentiates macrophage and neutrophil accumulation from the BM and blood to local inflammatory colon tissue in acute ulcerative colitis. 

### 3.4. Dex Treatment Potentiated Mucosal Expression of Pro-Inflammatory Cytokines and Chemokines

**Figure 3:**
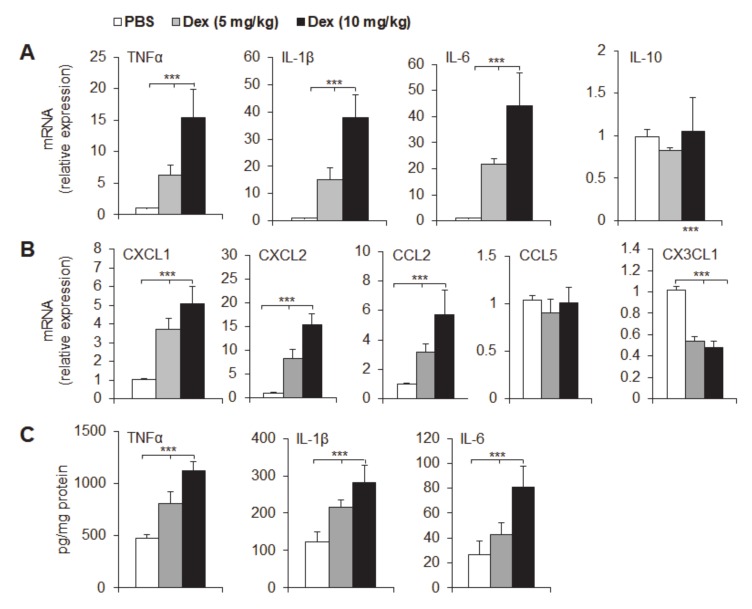
Dex promotes inflammatory cytokine production in acute ulcerative colitis mice. The indicated cytokine mRNA expression (**A**,**B**) was determined by qPCR, and the levels in the control group (phosphate buffered saline; PBS) were set to 1. (**C**) The indicated mucosal cytokine concentrations were measured by ELISA and are expressed as pg/mg total protein. Representative results are based on one of three or four independent experiments performed with similar results. The data are presented as the mean ± SD (*n* = 3–5 mice per group). Statistical significance was measured by one-way ANOVA. ***P < 0.001, compared with the indicated groups.

A variety of pro-inflammatory cytokines and chemokines are known to mediate intestinal inflammation, and we investigated cytokine and chemokine expression in colon samples from Dex-treated and control mice in acute DSS-induced colitis by qPCR (Figure 3a,b) and ELISA (Figure 3c). Dex treatment strongly upregulated the expression of the cytokines TNFα, IL-1β and IL-6 but not IL-10 in the course of DSS colitis compared with that in the control group (Figure 3a,c). These results suggest that Dex treatment significantly exacerbates acute colon inflammation and potentiates inflammatory cytokine secretion in local inflammatory colon tissue. We next determined the mRNA expression of key chemokines that are known to regulate the migration of neutrophils and monocytes/macrophages (Figure 3b). CXCL1 and CXCL2 are potential neutrophil recruitment chemokines that were significantly increased in Dex-treated mouse inflammatory mucosal tissue. CCL2 (MCP-1) and CX3CL1 (Fractalkine), but not CCL5 (Rantes), are potential monocyte chemoattractants that were also significantly upregulated in Dex-treated inflammatory mucosal tissue compared to that in control groups. These data are consistent with the increased accumulation of neutrophils and monocytes/macrophages in inflammatory mucosal colon tissue in acute Dex-treated colitis mice. 

### 3.5. The Microbiota Is Not Required for Dex-Induced Severe Acute Ulcerative Colitis

**Figure 4:**
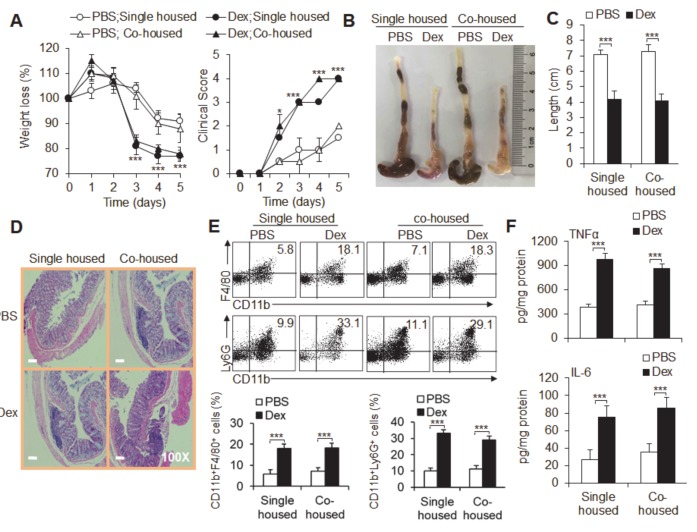
Reciprocal exchange in gut microbiota is not associated with Dex-potentiated acute experimental ulcerative colitis. The effects of dexamethasone (Dex) in dextran sulfate sodium (DSS)-induced mice were observed in separately housed (single-housed) and cohoused mice. Weight loss and disease clinical scores (**A**) are shown. Colon lengths (**B**,**C**) and pathological histological photos (**D**) are shown. (**E**) The flow cytometry staining of the indicated cells in the colons of acute colitis mice is shown. Representative flow cytometry figures are shown (upper), and the data are summarized (lower). (**F**) The mucosal cytokine concentrations of TNFα and IL-6 were measured by ELISA and are expressed as pg/mg total protein. Representative results are based on one of three or four independent experiments performed with similar results. The data are presented as the mean ± SD (*n* = 4 mice per group). Statistical significance was measured by one-way ANOVA for comparisons among multiple groups and Student’s unpaired *t* test for comparisons between two groups. *P < 0.05 and ***P < 0.001, compared with the indicated groups.

To confirm the effect of the microbiota on acute ulcerative colitis, we established acute experimental colitis by cohousing vehicle (PBS)-treated and Dex-treated colitis mice and comparing them with single-housed colitis mice. The mice were treated with vehicle or Dex for at least 6 weeks before and throughout these experiments. Although Dex-treatment still significantly exacerbated colitis, as evidenced by significantly lower body weight, a reduced DAI score and severe destructive macroscopic and histological pathological alterations as described above, the symptoms, pathological changes, inflammatory immune cell infiltration and inflammatory cytokine TNFα and IL-6 production in colitis showed no significant differences between cohoused and single-housed colitis mice (Figure 4a–f). These results suggest that reciprocal exchanges of different mouse microbiota did not play a critical role in Dex-induced severe acute experimental ulcerative colitis. 

### 3.6. mTOR, but not GR, Is Required for Dex-Induced Severe Ulcerative Colitis

Glucocorticoids usually play a regulatory role through their receptor GR. We therefore used flow cytometry (Figure S3a) or immunoblotting (Figure S3b) to determine the expression of GR in the inflammatory colonic cells of DSS colitis mice that were treated with PBS or Dex. However, there was no significant difference in GR expression in the colon cells between the Dex-treated and control groups, and there was also no significant difference in GR expression in the cytoplasm or nuclei of the colon cells between the two groups. 

Previous studies have shown that mTOR is critical for colitis and that p-S6 (an mTOR downstream target) is significantly upregulated in Dex-treated CD45^-^ colonic epithelial cells. We next investigated the expression of other mTOR signaling molecules. p-S6 was significantly upregulated in CD45^-^ cells from inflammatory colon tissue in DSS-induced acute colitis, as shown by flow cytometry (Figure S4a), and in colonic CD45^-^ epithelial cells, as shown by immunoblotting (Figure S4b). Additionally, different doses of Dex significantly upregulated p-mTOR and p-AKT (Thr308), which are downstream targets of mTORC1, but not p-AKT (Ser473), which is a downstream target of mTORC2, in CD45^-^ cells from colon tissue in DSS colitis mice (Figure S4c). These data suggest that mTORC1, but not mTORC2, in epithelial cells is critically required for acute DSS-induced ulcerative colitis.

**Figure 5:**
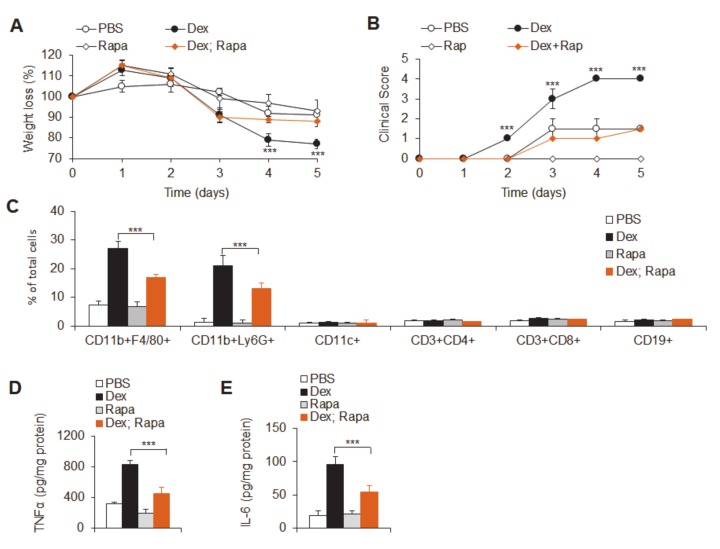
Rapamycin treatment alleviated Dex-potentiated DSS-induced ulcerative colitis. Dextran sulfate sodium (DSS)-induced mice were intraperitoneally (i.p.) injected with different doses of dexamethasone (Dex) (5 or 10 mg kg) and rapamycin (Rapa; 3 mg/kg). Weight loss (**A**) and disease clinical scores (**B**) are summarized. (**C**) The flow cytometry staining of the indicated cells in the colons of acute colitis mice is shown, and the data are summarized. (**D**,**E**) The indicated mucosal cytokine concentrations were measured by ELISA and are expressed as pg/mg total protein. Representative results are based on one of three or four independent experiments performed with similar results. The data are presented as the mean ± SD (*n* = 3–5 mice per group). Statistical significance was measured by one-way ANOVA. ***P < 0.001, compared with the indicated groups.

To confirm the role of mTOR in Dex-exacerbated experimental colitis, acute experimental ulcerative colitis was induced and then treated with Dex or rapamycin (a specific mTOR signal inhibitor, Rapa) or Rapa combined treatment with Dex, to observe the changes in acute experimental colitis. Consistent with the above results, Dex treatment significantly reduced the weights of mice, shortened the length of the inflamed colon, induced severe histopathological damage and resulted in a large amount of neutrophil and macrophage infiltration and pro-inflammatory cytokine secretion (Figure 5a–e). Rapa combined with Dex treatment significantly alleviated the symptoms of colitis, histopathological pathological damage, innate neutrophil and macrophage infiltration and pro-inflammatory cytokine secretion, although rapamycin treatment alone had no obvious effects (Figure 5a–e). These data strongly suggest that mTOR signaling is required for the Dex-mediated exacerbation of acute ulcerative colitis.

**Figure 6:**
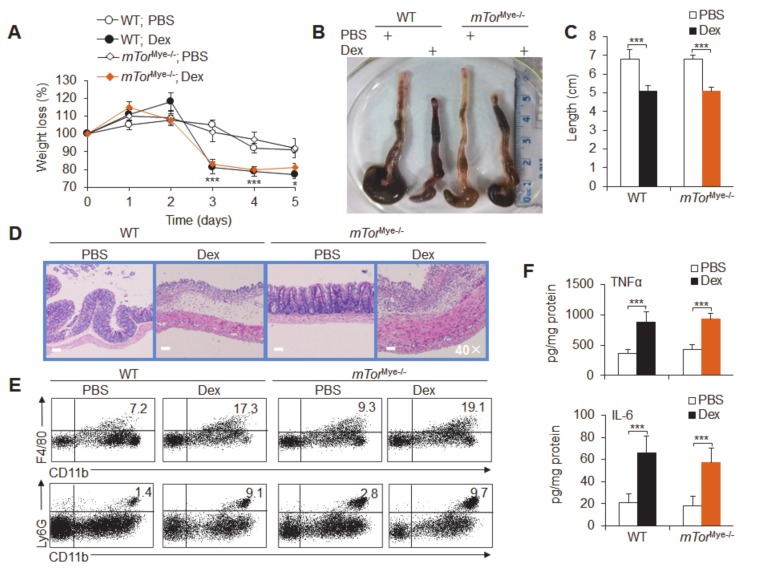
Macrophage/neutrophil mTOR is not required for Dex-potentiated acute experimental ulcerative colitis. The effects of mTOR in neutrophils and macrophages were observed in dexamethasone (Dex)-potentiated *mTor*^Mye-/-^ and wild-type (WT) acute colitis mice. Weight loss (**A**), colon lengths (**B**,**C**) and pathological histological photos (**D**) are shown. (**E**) The flow cytometry staining of infiltrating inflammatory cells in the colonic tissue of acute colitis mice is shown. (**F**) The mucosal concentrations of the cytokines TNFα and IL-6 were measured by ELISA and are expressed as pg/mg total protein. Representative results are based on one of three or four independent experiments performed with similar results. The data are presented as the mean ± SD (*n* = 4 mice per group). Statistical significance was measured by one-way ANOVA for comparisons among multiple groups and Student’s unpaired *t* test for comparisons between two groups. *P < 0.05 and ***P < 0.001, compared with the indicated groups.

Then, we investigated the effects of mTOR in myeloid cells and epithelial cells with conditional knockout mice that were subjected to Dex-treated acute colitis. We crossed *mTor*^fl/fl^ and *Lyz-Cre* mice to conditionally delete *mTor* gene expression in myeloid cells, including macrophages and neutrophils; these mice were called *mTor*^Mye−/−^ thereafter (Figure S5). Although Dex treatment caused significantly severe colitis, mTOR signaling deficiency in myeloid cells did not significantly alter the course of DSS-induced colitis, including weight loss, changes in colon length, pathological damage, inflammatory immune cell infiltration and the production of the inflammatory cytokines TNFα and IL-6 (Figure 6a–f). These results suggest that myeloid mTOR is not involved in Dex-induced severe colitis.

**Figure 7:**
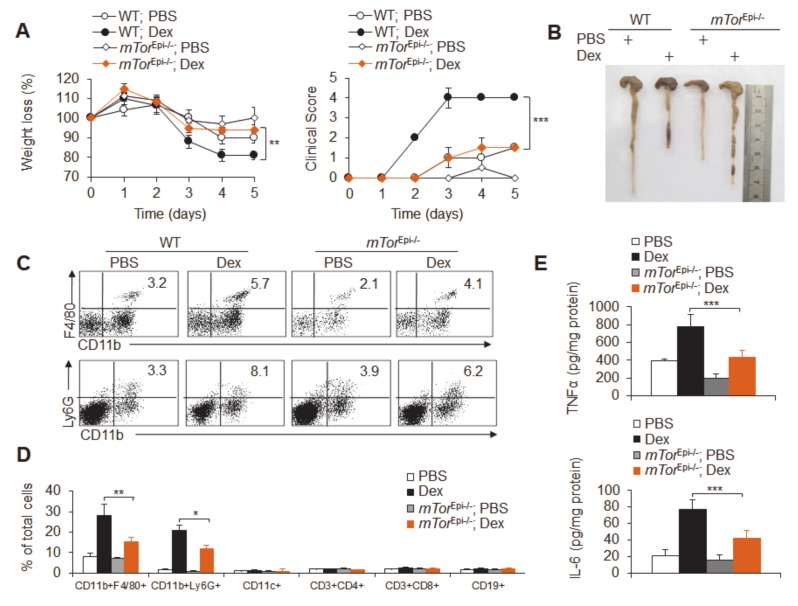
Intestinal epithelial cell-mediated mTOR signaling is critical for Dex-potentiated acute experimental ulcerative colitis. The effects of mTOR in epithelial cells were observed in dexamethasone (Dex)-potentiated *mTor*^Epi−/−^ and wild-type (WT) acute colitis mice. Weight loss (**A**) and colon lengths (**B**) are shown. (**C**,**D**) The flow cytometry staining of infiltrating inflammatory cells in the colonic tissue of acute colitis mice is shown; a representative figure is shown (**C**), and the data are summarized (**D**). (**E**) The concentrations of the mucosal cytokines TNFα and IL-6 were measured by ELISA and are expressed as pg/mg total protein. Representative results are based on one of three or four independent experiments performed with similar results. The data are presented as the mean ± SD (*n* = 3–4 mice per group). Statistical significance was measured by one-way ANOVA. *P < 0.05, **P < 0.01 and ***P < 0.001, compared with the indicated groups.

Furthermore, we crossed *mTor^fl/fl^* and *Vil-Cre* mice to conditionally delete *mTor* gene expression in intestinal epithelial cells; these were called *mTor*^Epi−/−^ mice. Interestingly, mTOR deficiency in epithelial cells partially, but significantly, recovered the severe course of colitis caused by Dex, including weight loss, changes in clinical score, changes in colon length, pathological damage, inflammatory cell infiltration and pro-inflammatory cytokine production (Figure 7a–e and Figure S6a,b). Thus, intestinal epithelial cell mTOR is critical for Dex-induced severe ulcerative colitis.

### 3.7. The Dex-mTOR Signal Axis Potentiated Colitis-Associated Cancer

**Figure 8:**
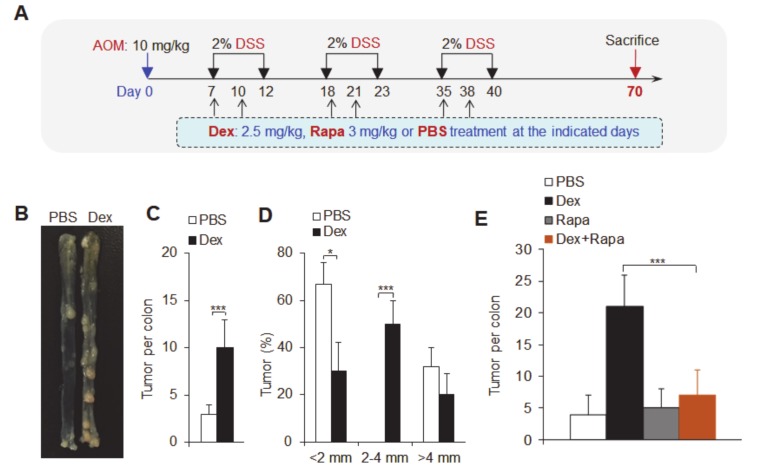
Dex promotes colitis-associated tumorigenesis. (**A**) The structural schematics of dexamethasone (Dex)-treated mice and the induction of colitis-associated tumorigenesis. (**B**) Colon tumors in control (phosphate buffer saline; PBS) or Dex-treated mice 70 days after the injection of azoxymethane (AOM). (**C**,**D**) The number and size of colon tumors in control or Dex-treated mice. (**E**) The number of colon tumors with the indicated treatments. Representative results are based on one of three independent experiments performed with similar results. The data are presented as the mean ± SD (*n* = 10–12 mice per group). Statistical significance was measured by one-way ANOVA for comparisons among multiple groups and Student’s unpaired *t* test for comparisons between two groups. *P < 0.05 and ***P < 0.001, compared with the indicated groups.

To further test the critical role of the Dex-mTOR signaling axis in colitis-associated cancer, we established a colitis-associated cancer mouse model by using the combined administration of the genotoxic agent azoxymethane (AOM) and DSS, as described in Figure 8a. At 70 days after treatment with Dex and vehicle (PBS) with the indicated dose, we found that Dex treatment significantly increased the number of tumors in the colon (Figure 8b,c), mainly increasing the number of 2–4 mm-sized tumors and decreasing the number of tumors smaller than 2 mm, but there were no significant effects on the number of larger tumors (Figure 8d). Combination treatment with the mTOR inhibitor rapamycin significantly recovered the tumor numbers (Figure 8e and Figure S7). These data suggest that Dex-mTOR signaling is required for colitis-associated cancer.

## 4. Discussion

Synthetic glucocorticoid immunosuppressants, especially Dex, have been widely used in treating inflammatory disorders and are well known for their effects on the immune system [7,28,48,49,50]. It has been suggested that GCs regulate a variety of different immune cell activities [51]. Dex alters the phenotype and function of dendritic cells (DCs), rendering them tolerogenic [52]. Moreover, Dex has been regarded as a tolerogenic adjuvant through the in vivo selection of tolerogenic macrophages [53]. Therefore, glucocorticoids have been widely used in clinical practice, especially in acute severe inflammatory diseases, and are often used as the first-line treatment for acute severe inflammation. While glucocorticoids are effective in the treatment of severe inflammatory disorders, there are sometimes complications of colitis for unknown reasons. In this study, our data showed that glucocorticoid therapy contributes to the onset and development of acute experimental ulcerative colitis and colitis-associated cancer in mice (Figure S8).

Acute ulcerative colitis results in the accumulation of innate immune cells, such as neutrophils and macrophages, and the enhanced secretion of pro-inflammatory cytokines, such as TNFα. Therefore, glucocorticoids are also used as a first-line emergency treatment drug in the clinic. If treatment is ineffective or exacerbates the condition, immunosuppressive agents such as cyclosporine A or the blocking of the secretion of the pro-inflammatory cytokine TNFα with infliximab are used for emergency intravenous infusion treatment. Partial colectomy is considered if treatment is no longer effective [2,4,7,54,55,56]. It is important to further summarize these clinical studies, and the glucocorticoid treatment of acute ulcerative colitis, especially in the development of acute ulcerative colitis, is often ineffective or even detrimental. However, glucocorticoids combined with immunosuppressants and TNFα blockade are often effective in the clinic [7,12,13,14,15,16,17,18,54]. However, the theoretical basis of this phenomenon is not clear. Herein, this study showed that in the early stages of acute experimental ulcerative colitis, glucocorticoid therapy significantly exacerbated the occurrence of acute ulcerative colitis. The mechanism is mainly the upregulation of mTOR signaling in the intestinal epithelial cells but not in immune cells. The long-term use of glucocorticoids could promote the development of colitis-related cancers. The combination of glucocorticoids and mTOR signaling deficiency or the mTOR signaling inhibitor rapamycin significantly alleviated the formation of acute ulcerative colitis and chronic colitis-associated cancer in later stages (Figure S8). Thus, this study provides a theoretical basis for the effectiveness of the current combined immunosuppressive regimen in patients with acute ulcerative colitis. Moreover, these data also showed that if glucocorticoids are used in the treatment of acute severe inflammation, the side effects of acute colitis could be avoided by blocking mTOR signaling in intestinal epithelial cells.

mTOR is a Ser/Thr kinase that affects many aspects of cellular function, including immunity [57,58]. mTORC1 is the core component of mTOR, which acts downstream of the PI3K-Akt pathway to phosphorylate p70S6 kinase (p70S6K) in a rapamycin-sensitive fashion [43,59,60]. By contrast, mTORC2 induces Akt by Ser473 phosphorylation [61,62,63,64]. Using both pharmaceutical and genetic strategies to investigate the consequences of mTORC1 inhibition in 2,4,6-trinitrobenzene sulfonic acid (TNBS)- and DSS-induced experimental ulcerative colitis mouse models showed an increased susceptibility and mortality of mice and that mTORC1 is essential for the proliferation of intestinal epithelial cells to protect against inflammatory bowel diseases [47]. Another study showed that mTORC1-deficient DCs displayed an increased susceptibility to DSS-induced colitis, suggesting that mTORC1 is essential in intestinal CD11c^+^CD11b^+^ DCs and is critical for regulating intestinal homeostasis by promoting IL-10 production [44]. Therefore, mTOR signaling regulates intestinal epithelial cells or affects the function of immune cells in colitis. Moreover, there is a reciprocal regulatory relationship between mTOR signaling and glucocorticoids in controlling the function of innate immune cells. As recently reported, the mTOR pathway interferes with glucocorticoid signaling in the regulation of innate immune cell functions [29,65]. The inhibition of mTOR with rapamycin or Torin1 reduces the anti-inflammatory potency of glucocorticoids in both human monocytes and myeloid DCs. Glucocorticoids did not suppress nuclear factor-kappa B and JNK activation, the expression of pro-inflammatory cytokines or the promotion of Th1 responses when mTOR was inhibited. Additionally, the long-term activation of monocytes with LPS enhanced the expression of TSC2, the principle negative regulator of mTOR, whereas Dex blocked TSC2 expression and reestablished mTOR activation. Consistently, our study showed that although Dex treatment did not alter the expression of GR in intestinal cells, Dex treatment upregulated mTOR activation, mostly in intestinal epithelial cells, in acute experimental ulcerative colitis. Furthermore, our study also showed that mTOR signaling in intestinal epithelial cells is critical for maintaining immune homeostasis in acute DSS-induced ulcerative colitis. In addition, the combination of Dex treatment with the blockade of mTOR signaling in intestinal epithelial cells significantly recovered the Dex-induced exacerbation of colitis. Glucocorticoids are widely used in the treatment of acute severe clinical diseases. In this way, targeting mTOR signaling in epithelial cells can effectively reduce the incidence of acute colitis complications caused by clinical glucocorticoids in the treatment of other acute severe inflammatory diseases.

Although this study identified that glucocorticoid treatment can aggravate acute ulcerative colitis in mice, it only observed the onset and development of acute ulcerative colitis in mice, and found that glucocorticoid-induced mTOR signaling in epithelial cells is required in the early stages of acute ulcerative colitis by modulating the dynamics of innate immune cell recruitment and activation. However, it is still unclear whether there is a regulatory or aggravating effect on mice already suffering from acute and chronic ulcerative colitis or other colonic diseases, which needs further study. This study also showed that glucocorticoids have regulatory effects in the colitis-associated cancer mouse model, but the specific regulatory characteristics are not clear. The results in the experimental mouse ulcerative colitis model also need to be further investigated in clinical patients.

In summary, it has been shown that targeting mTOR signaling in intestinal epithelial cells controls the recruitment and activation of innate inflammatory macrophages and neutrophils, thereby contributing to the onset and development of acute experimental ulcerative colitis and colitis-associated cancer. Thus, our results define the essential nature of glucocorticoid treatments and the mTOR pathway as the mechanism by which intestinal epithelial cells regulate the recruitment and activation of innate inflammatory cells, and targeting intestinal epithelial cells is a potential approach to preventing glucocorticoids from exacerbating acute colitis and colitis-associated cancer complications in the treatment of other severe inflammatory diseases.

## Figures and Tables

**Table 1:** The primer sequences used for the real-time PCR assays.

Gene Names	Primer Sequences (5ʹ-3ʹ)
HPRT	Forward: AGTACAGCCCCAAAATGGTTAAG
	Reverse: CTTAGGCTTTGTATTTGGCTTTTC
IL-1β	Forward: TGGGAAACAACAGTGGTCAGG
	Reverse: CCATCAGAGGCAAGGAGGAA
IL-6	Forward: GCAATGGCAATTCTGATTGTATG
	Reverse: CCAGTGCCTCTTTGCTGCTTTC
TNFα	Forward: GAGTGACAAGCCTGTAGCC
	Reverse: CTCCTGGTATGAGATAGCAAA
IL-10	Forward: GCTCTTACTGACTGGCATGAG
	Reverse: CAA TACCATTGACCTGCCGAT
CXCL1	Forward: GCACCCAAACCGAAGTCATAG
	Reverse: AGAAGCCAGCGTTCACCAGA
CXCL2	Forward: GCCCAGACAGAAGTCATAGCC
	Reverse: CTCCTCCTTTCCAGGTCAGTTA
CX3CL1	Forward: CGCAATCATCTTGGAGACGA
	Reverse: GTGCCGCCATTTCGAGTTA
CCL1	Forward: TCAGCCAGATGCAGTTAACGC
	Reverse: TGATCCTCTTGTAGCTCTCCAGC
CCL5	Forward: GATGGACATAGAGGACACAACT
	Reverse: TGGGACGGCAGATCTGAGGG

## Supplementary Materials

The following are available online at https://www.mdpi.com/2072-6694/12/4/945/s1, Figure S1: Dex upregulates mTOR in inflammatory colon tissue, Figure S2: Dex upregulates mTOR in acute experimental ulcerative colitis, Figure S3: Dex treatment could not alter GR signals in colonic tissue of acute colitis, Figure S4: Dex treatment upregulates mTOR (C1) signals in epithelia cells of colonic tissue of acute ulcerative colitis, Figure S5: Expressions of p-mTOR in myeloid cells in DSS-induced colitis in mice, Figure S6: mTOR deficiency in epithelial cells recovered the Dex-promoted the neutrophil and macrophage recruitment, Figure S7: Expressions of p-mTOR in the colonic tissue of colitis-associated tumorigenesis in mice, Figure S8: Dex promotes the onsets of acute experimental colitis by upregulating mTOR signaling in epithelia cells, Figure S9: Original western blots for images used in main figures.
