# Supplementary material for: Glucocorticoids Promote the Onset of Acute Experimental Colitis and Cancer by Upregulating mTOR Signaling in Intestinal Epithelial Cells
Source: Cancers (Basel). 2020 Apr 11;12(4):945. doi: 10.3390/cancers12040945 (PMC7254274; doi:10.3390/cancers12040945)
Supplement: Supplementary file 1 [file cancers-12-00945-s001.pdf]

Supplementary Materials: Glucocorticoids promote the onset of acute experimental colitis by up-regulating mTOR signal in intestinal epithelial cells

Zhengguo Zhang, Lin Dong, Anna Jia1, Xi Chen, Qiuli Yang, Yufei Wang, Yuexin Wang, Ruichen Liu, Yejin Cao, Ying He, Yujing Bi and Guangwei Liu

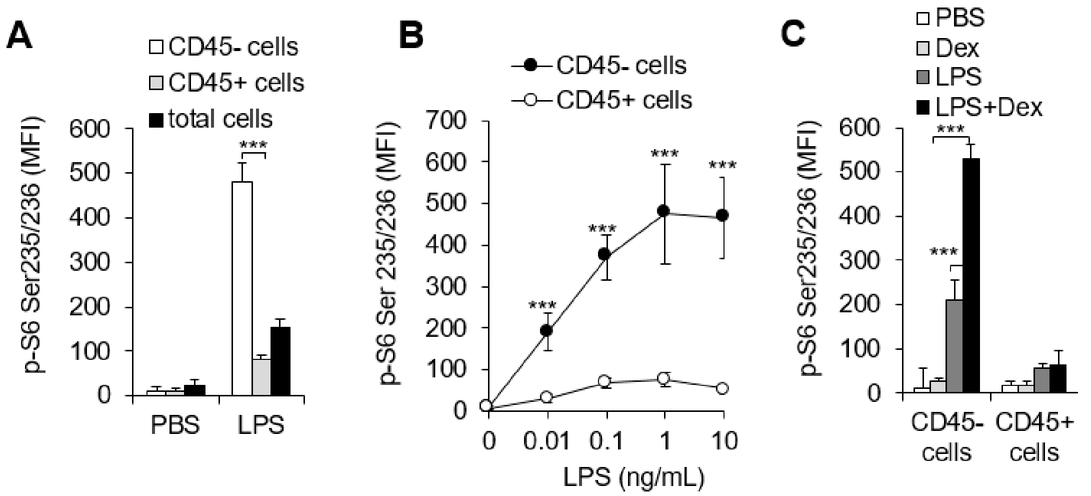

Figure S1. Dex upregulates mTOR in inflammatory colon tissue. (**A**) CD45+, CD45- cells and total cells isolated from colon tissue and treated as indicated, the p-S6 Ser 235/236 was determined with flow cytometry and the MFI of p-S6 Ser235/236 summarized. (**B**) MFI of p-S6 Ser 235/236 in CD45+ and CD45- cells isolated from colon and stimulated with different dose of LPS as indicated. (C) MFI of p-S6 Ser235/236 in CD45+ and CD45- cells isolated from colon and treated as indicated. Data are representative of three to four independent experiments (n=4 mice per group). ***P<0.001, compared with the indicated groups.

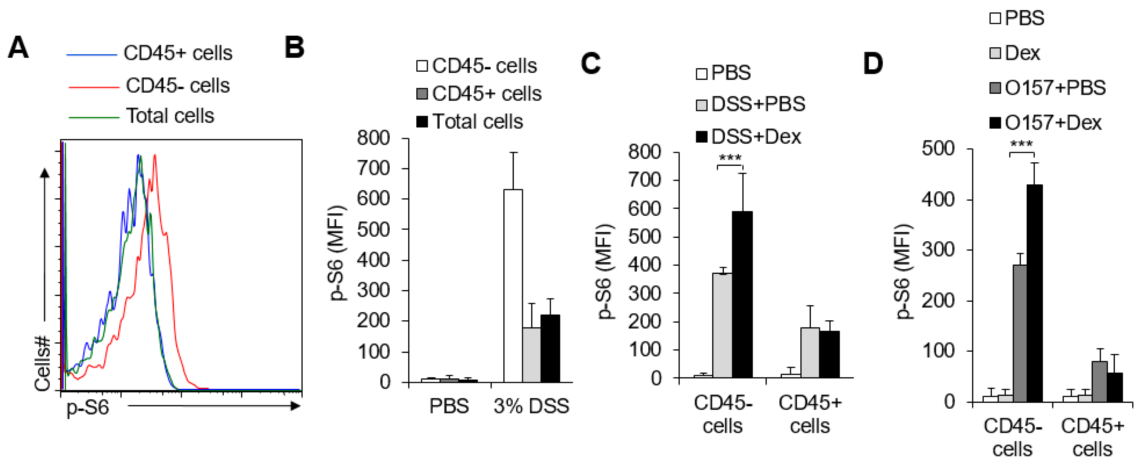

Figure S2. Dex upregulates mTOR in acute experimental ulcerative colitis. (**A** and **B**) 3% DSS induced colitis for 5 days and $CD45^+$ cells, $CD45^-$ cells and total colon cells were isolated and the expression of p-S6 Ser235/236 was determined with flow cytometry (**A**) and the MFI of p-S6 Ser235/236 summarized (**B**). (**C** and **D**) Mouse colitis was induced by 3% DSS (**C**) or O157 *E.coli* infection (**D**) in combination with indicated treatment for 5 days, the $CD45^-$ and $CD45^+$ cells isolated from indicated colon and the MFI of p-S6 Ser235/236 was determined and summarized. Data are representative of three to three independent experiments (n=4 mice per group). ****P*<0.001, compared with the indicated groups.

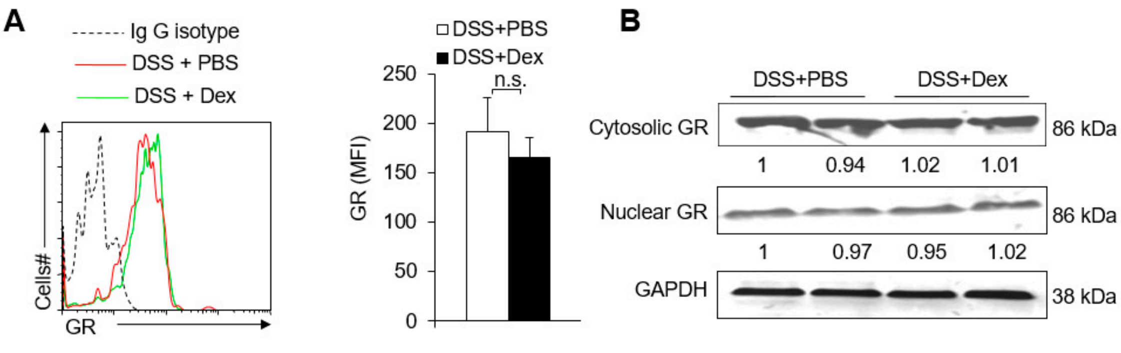

Figure S3. Dex treatment could not alter GR signals in colonic tissue of acute colitis. The single-cell suspensions isolated from colon tissue in mice treated with 3% DSS for 5 days in combination with Dex or vehicle (PBS) i.p. injection daily. The MFI of GR (A) measured by flow cytometry and typical figure shown (left) and data summarized (right). And the expressions of indicated signals were determined by Western blot (B). The expression levels of indicate signal were normalized to the control group. The fold changes between indicated signal and GAPDH were calculated and the expression in DSS+PBS left group in this case was set as 1. Data are representative of three to four independent experiments (n=4-5 mice per group). n.s., not significant.

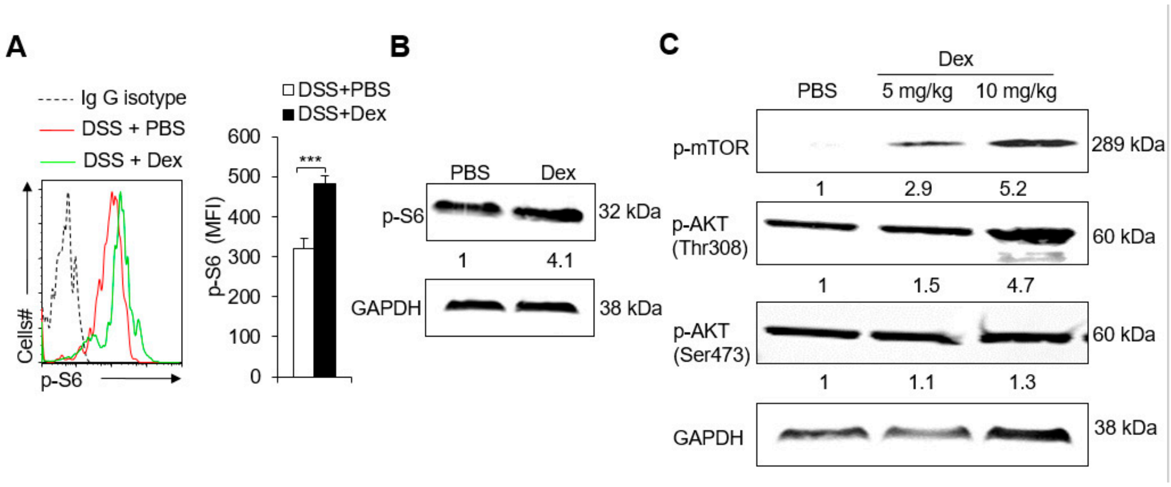

Figure S4. Dex treatment upregulates mTOR (C1) signals in epithelia cells of colonic tissue of acute ulcerative colitis. The single-cell suspensions derived from mice treated with 3% DSS for 5 days in combination with Dex or vehicle (PBS) i.p. injection daily. (**A**) The MFI of p-S6 Ser235/236 in CD45^{-} cells was measured by flow cytometry and typical figure shown (left) and data summarized (right). (**B-C**) CD45^{-} cells isolated from single-cell suspensions of colon tissue and the expressions of p-S6 Ser235/236 (**B**) and indicated signals (**C**) were determined by Western blot. The expression levels of indicate signal were normalized to the control group. The fold changes between indicated signal and GAPDH were calculated and the expression in PBS group in this case was set as 1. Data are representative of three to four independent experiments (n=4-5 mice per group). ***P<0.001, compared with the indicated groups.

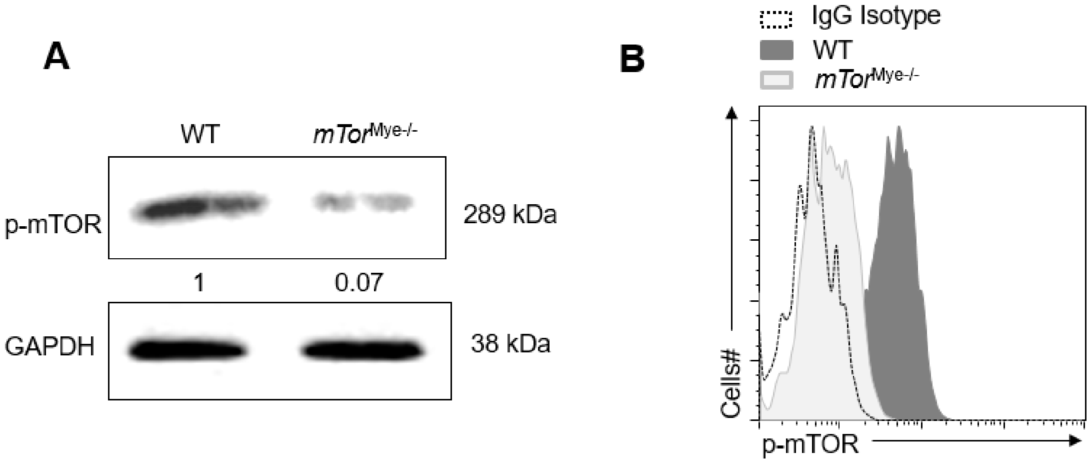

Figure S5. Expressions of p-mTOR in myeloid cells in DSS-induced colitis in mice. $CD11b^{+}$ myeloid cells were isolated from the single-cell suspensions of colonic tissue of DSS-induced colitis in *mTor*$^{Mye-/-}$ and WT mice. The expressions of indicated signals were determined by Western blot (**A**) and flow cytometry (**B**). The expression levels of p-mTOR in Western blot were normalized to the WT group. The fold changes between p-mTOR and GAPDH were calculated and the expression in WT group in this case was set as 1.

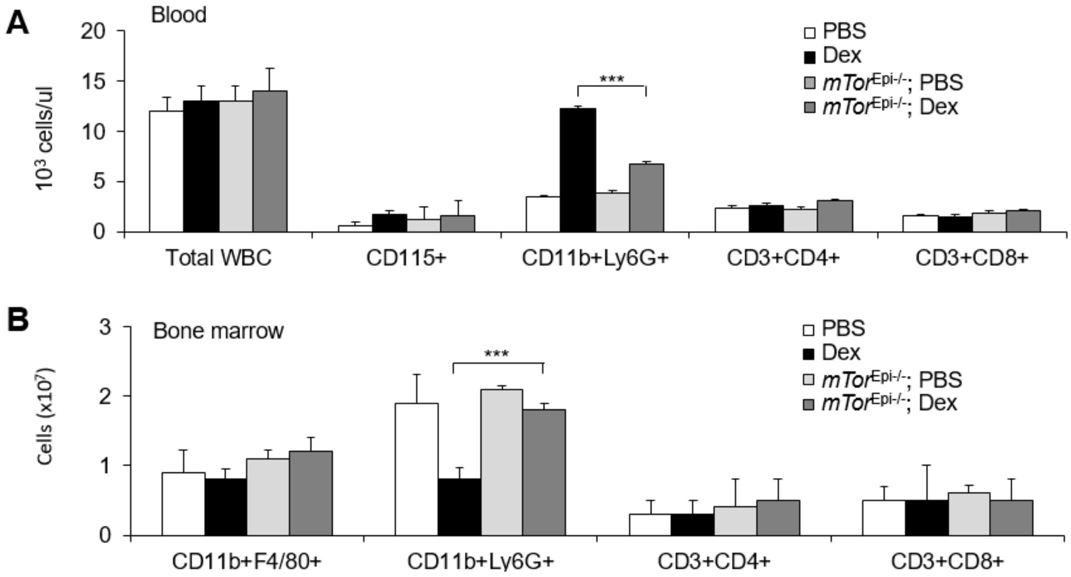

Figure S6. mTOR deficiency in epithelial cells recovered the Dex-promoted the neutrophil and macrophage recruitment. Relative proportions of macrophages ($CD11b^{+}F4/80^{+}$), neutrophils ($CD11b^{+}Ly6G^{+}$), DC ($CD11c^{+}$), $CD4^{+}$T cells ($CD3^{+}CD4^{+}$), $CD8^{+}$T cells ($CD3^{+}CD8^{+}$) and B cells ($CD19^{+}$) in single-cell suspensions derived from mice treated with 3% DSS for 5 days in *mTor*$^{Epi-/-}$ mice and WT mice combination with Dex or vehicle (PBS) i.p. injection daily. (**A**) The blood cells of mice were analyzed. The $CD11b^{+}CD115^{+}$ were used as marker of monocytes, the data summarized. (**B**) The bone marrow cells of mice were analyzed and the data summarized. Data are representative of three to four independent experiments (n=4-8 mice per group). ***P<0.001, compared with the indicated groups.

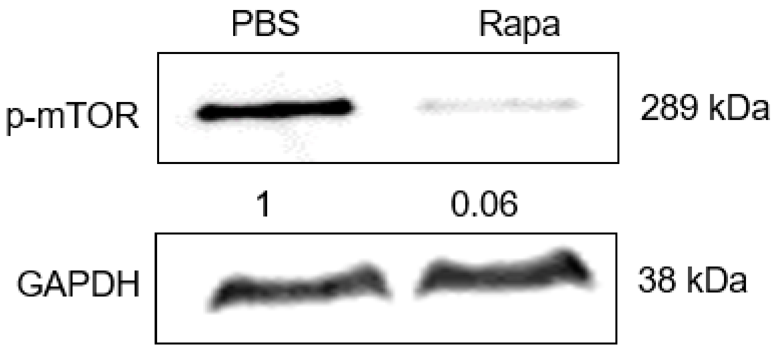

Figure S7. Expressions of p-mTOR in the colonic tissue of colitis-associated tumorigenesis in mice. Colonic tissue in PBS- or Rapa-treated mice 70 days after injection of AOM. The expressions of indicated signals were determined by Western blot. The expression levels of p-mTOR were normalized to the control group. The fold changes between p-mTOR and GAPDH were calculated and the expression in PBS groups in this case was set as 1.

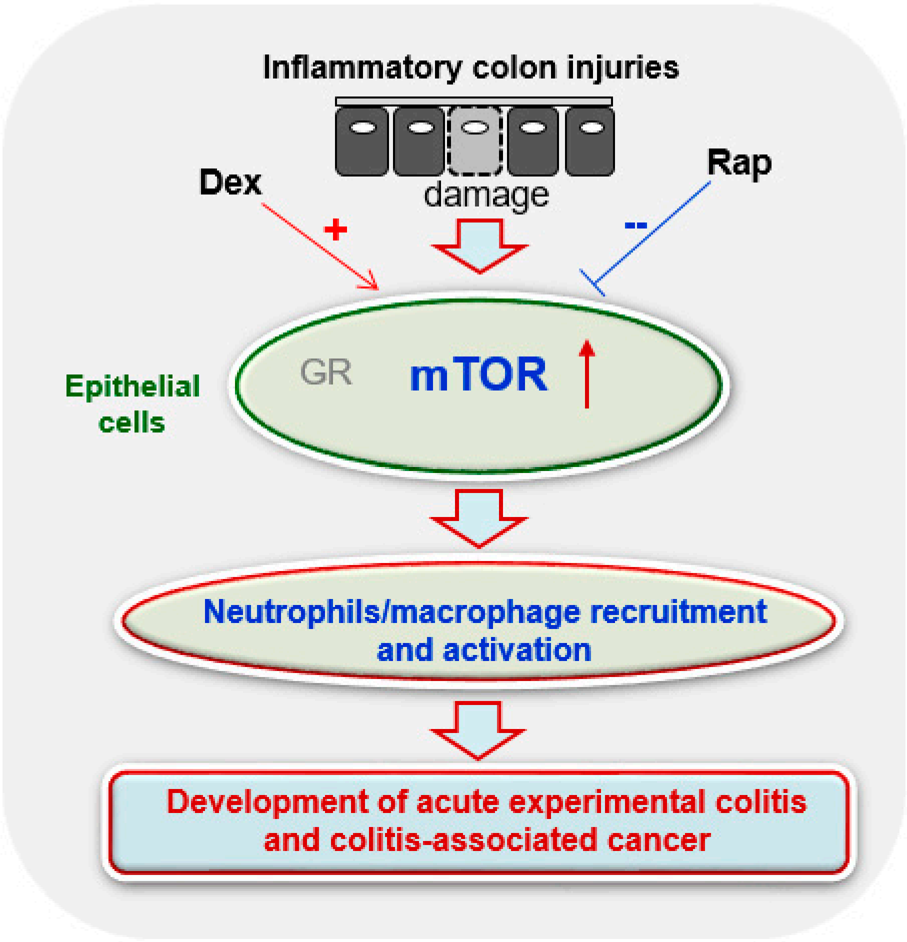

Figure S8. Dex promotes the onsets of acute experimental colitis by upregulating mTOR signaling in epithelia cells. Proposed model of how mTOR signal in epithelial cells response to Dex treatment and innate stimuli to regulate the function and recruitment of innate immune cells in acute experimental colitis.

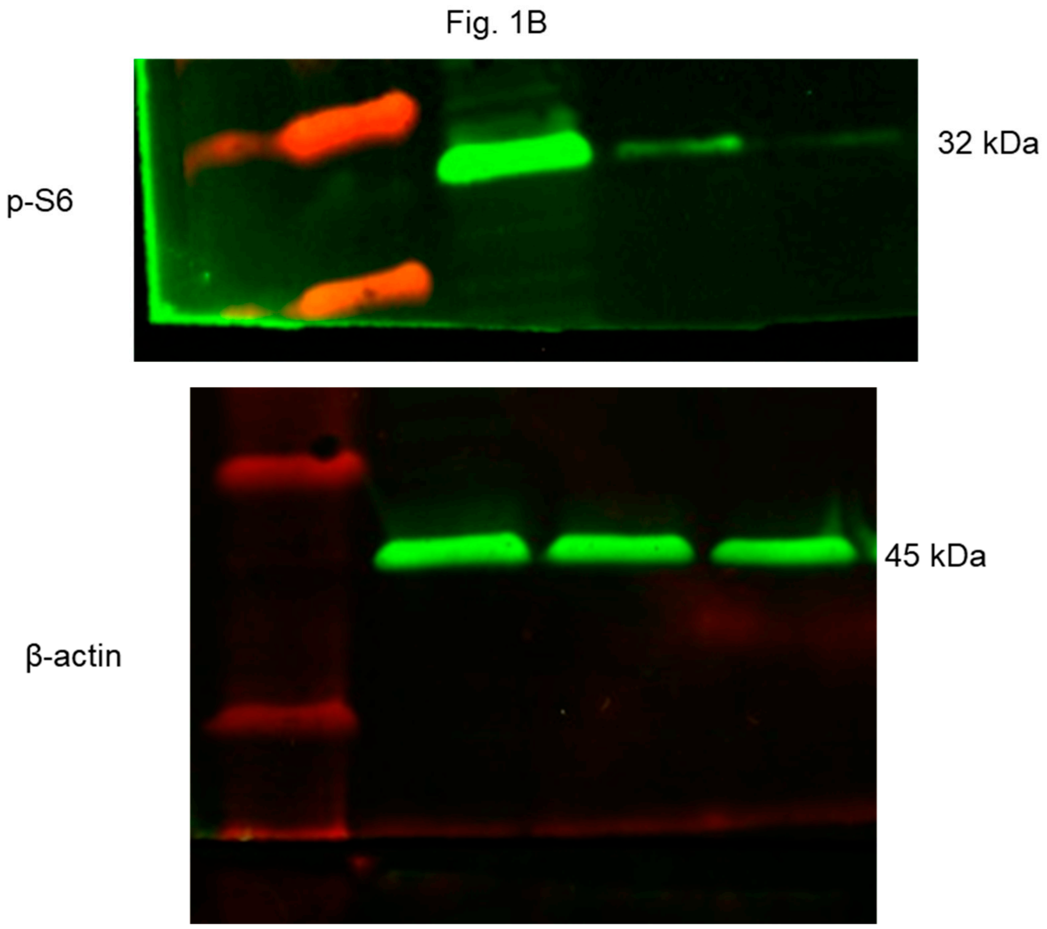

Figure S9. Original western blots for images used in the main figures. Each antibody produced a clear band at the expected size. Blocks indicate the specific bands used in the main figure 1B.

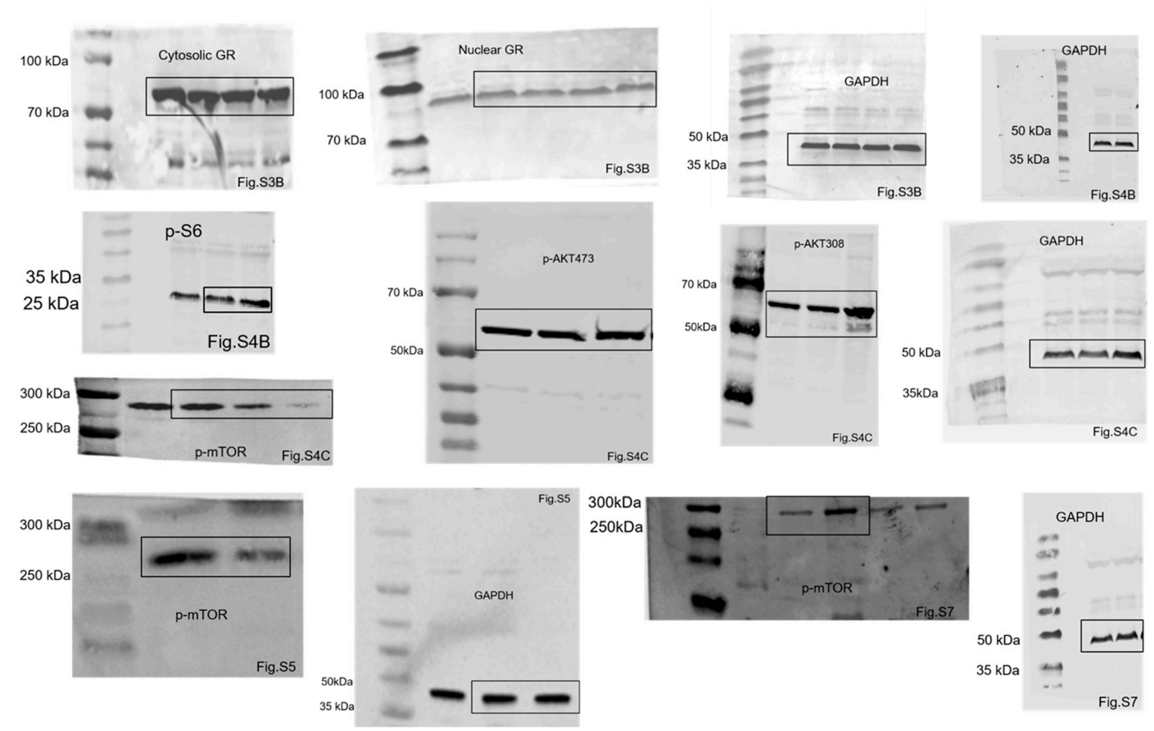

Figure S10. Original western blots for images used in the supplementary figures. Each antibody produced a clear band at the expected size. Blocks indicate the specific bands used in the Figure S3B, Figure S4B, Figure S4C, Figure S5 and Figure S7.

© 2020 by the authors. Submitted for possible open access publication under the terms and conditions of the Creative Commons Attribution (CC BY) license (http://creativecommons.org/licenses/by/4.0/).
